# Ultra-accurate Duplex Sequencing for the assessment of pretreatment *ABL1* kinase domain mutations in Ph+ ALL

**DOI:** 10.1038/s41408-020-0329-y

**Published:** 2020-05-26

**Authors:** Nicholas J. Short, Hagop Kantarjian, Rashmi Kanagal-Shamanna, Koji Sasaki, Farhad Ravandi, Jorge Cortes, Marina Konopleva, Ghayas C. Issa, Steven M. Kornblau, Guillermo Garcia-Manero, Rebecca Garris, Jake Higgins, Gabriel Pratt, Lindsey N. Williams, Charles C. Valentine, Victor M. Rivera, Justin Pritchard, Jesse J. Salk, Jerald Radich, Elias Jabbour

**Affiliations:** 1 The University of Texas MD Anderson Cancer Center, Houston, TX USA; 2 TwinStrand Biosciences Inc., Seattle, WA USA; 3 ARIAD Pharmaceuticals, Inc., Cambridge, MA USA; 4 Pennsylvania State University, University Park, PA USA; 5 Fred Hutchinson Cancer Research Center, Seattle, WA USA

**Keywords:** Translational research, Cancer genetics

## Abstract

Mutations of *ABL1* are the dominant mechanism of relapse in Philadelphia chromosome-positive acute lymphoblastic leukemia (Ph + ALL). We performed highly accurate Duplex Sequencing of exons 4–10 of *ABL1* on bone marrow or peripheral blood samples from 63 adult patients with previously untreated Ph + ALL who received induction with intensive chemotherapy plus a BCR-ABL1 TKI. We identified *ABL1* mutations prior to BCR-ABL1 TKI exposure in 78% of patients. However, these mutations were generally present at extremely low levels (median variant allelic frequency 0.008% [range, 0.004%–3.71%] and did not clonally expand and lead to relapse in any patient, even when the pretreatment mutation was known to confer resistance to the TKI received. In relapse samples harboring a TKI-resistant *ABL1* mutation, the corresponding mutation could not be detected pretreatment, despite validated sequencing sensitivity of Duplex Sequencing down to 0.005%. In samples under the selective pressure of ongoing TKI therapy, we detected low-level, emerging resistance mutations up to 5 months prior to relapse. These findings suggest that pretreatment *ABL1* mutation assessment should not guide upfront TKI selection in Ph + ALL, although serial testing while on TKI therapy may allow for early detection of clinically actionable resistant clones.

## Introduction

Philadelphia chromosome-positive acute lymphoblastic leukemia (Ph + ALL) is an aggressive form of leukemia that is associated with a relatively short duration of response with chemotherapy alone^1^. Outcomes are significantly improved with the integration of a BCR-ABL1-targeted tyrosine kinase inhibitor (TKI) into treatment regimens, with 5-year overall survival (OS) rates of 40–50% observed in patients treated with first- or second-generation TKIs combined with cytotoxic chemotherapy^2,3^. Despite the relative improvement in outcomes with the addition of TKIs, relapses are still common. The dominant mechanism of treatment failure in these patients is the development of mutations in the kinase domain (KD) of *ABL1* that confer TKI resistance (TKI-R) and ultimately lead to relapse. In Ph + leukemias, more than 60 different *ABL1* amino acid substitutions have been described in TKI-R cases^4^. In particular, T315I mutations lead to resistance of all first- and second-generation TKIs and are identified at the time of relapse in up to 75% of patients with Ph + ALL^2,5,6^. Some studies have suggested that low-abundance KD mutations in *ABL1*, including T315I mutations, may be present at diagnosis or early in treatment in up to 25% of patients with Ph + ALL^6–9^. The presence of pretreatment, clinically relevant subclonal mutations could have important implications for personalized therapy decisions in Ph + ALL, including the early incorporation of broad-spectrum BCR-ABL1 TKIs that target T315I or other *ABL1* resistance mutations for select patients where these are identified.

The accurate evaluation of low-level subclonal mutations in Ph + ALL and other malignancies is hindered by the limited sensitivity and/or specificity of available methodologies. For instance, reverse transcriptase polymerase chain reaction (RT-PCR) may introduce random errors and lead to false-positive results when assessing mutations at the level of RNA^10,11^. Similarly, due to artefactual errors occurring during PCR or sequencing steps, conventional next-generation sequencing (NGS) of genomic DNA (gDNA) cannot reliably detect subclonal mutations with a variant allelic frequency (VAF) < 1%. Given that previous reports have suggested that pretreatment TKI-R mutations may be present at levels as low 0.01%^8^, conventional NGS may miss a substantial proportion of true mutations and erroneously identify others. It is conceivable that technical limitations might explain some of the wide range of findings in this field over the last decade^6–9^.

To overcome the limitations of conventional mutation-detection methodologies, we used Duplex Sequencing to characterize the mutational spectrum of *ABL1* prior to TKI treatment and over the course of therapy. Duplex Sequencing is a powerful sequencing method that separately tags and sequences both strands of individual DNA molecules^12,13^. By comparing the nucleotide sequences of each strand of DNA and excluding sites of mismatches (artefactual errors) from analysis, Duplex Sequencing is able to identify true mutations with extremely high sensitivity and specificity, improving the accuracy of conventional NGS by >10,000-fold^13^. We postulated the higher sensitivity and specificity of Duplex Sequencing would more accurately detect and quantify low-level, pretreatment *ABL1* mutations compared to conventional techniques and could resolve historical uncertainties around the clinical importance of such mutations, thus providing important insights into optimal frontline TKI selection for patients with Ph + ALL.

## Methods

### Patients

We analyzed samples from adult patients with previously untreated Ph + ALL who received induction with hyperfractionated cyclophosphamide, vincristine, doxorubicin and dexamethasone alternating with methotrexate and high-dose cytarabine (hyper-CVAD regimen) plus a TKI at The University of Texas MD Anderson Cancer Center (UTMDACC) as previously described^2,3,14^. A total of 63 patients were selected for analysis based on availability of banked specimens. All patients provided informed consent for treatment according to institutional guidelines and the Declaration of Helsinki. This retrospective analysis of stored samples was approved by the Institutional Review Board of UTMDACC.

### Samples

Blood and/or bone marrow samples were collected prior to initiation of TKI treatment, between 1–6 months prior to relapse, and at the time of relapse. DNA was extracted from frozen cells or dried smears using a DNeasy Blood and Tissue kit (Qiagen) and quantified on a Qubit 3 Fluorometer with Qubit dsDNA High Sensitivity reagents (Invitrogen). DNA integrity was checked on a TapeStation 2100 (Agilent).

### Duplex Sequencing

Duplex Sequencing was performed under blinded conditions at TwinStrand Biosciences, Inc. Duplex Sequencing libraries were prepared from pretreatment, pre-relapse or relapse gDNA as previously described with minor modifications^15^. Briefly, up to 1000 ng of gDNA was sheared to a peak fragment size of 300 bp using a Covaris ME220 ultrasonicator (Covaris). Sheared DNA was end-repaired, A-tailed, and Duplex Adaptor-ligated using a KAPA HyperPrep Kit (Roche Sequencing) or a custom library prep kit (TwinStrand Biosciences, Inc.). Libraries were amplified with Illumina-compatible indexing primers. Amplified libraries underwent hybrid capture with 120-mer biotinylated oligonucleotide probes targeting *ABL1* exons 4–10, followed by PCR with P5/P7 primers. Owing to the small panel size, a second round of hybrid capture and PCR was performed to increase the on-target percentage^12^. Final libraries were quantified, normalized, pooled and sequenced on a NextSeq 500 (Illumina) with 151 bp paired-end reads. Sequencing reads were aligned to hs38DH and reads sharing common molecular tags with both strands represented were grouped into error-corrected Duplex Consensus Sequences (DCS) essentially as previously described^15^. Variants were called from DCS data with VarDictJava using custom high-stringency parameters.

Complementary DNA (cDNA) was synthesized using total RNA, random hexamer primers, dNTPs, magnesium with Superscript II reverse transcriptase (Invitrogen) and an RNase inhibitor (RNaseOUT) on ABI 9600/9700 thermocyclers. Second-strand synthesis was performed with a single *ABL1* exon 3 forward primer and one cycle of annealing and extension with KAPA HiFi HotStart ReadyMix 2×(Roche Sequencing). Duplex Sequencing library preparation with 10 ng of double-stranded cDNA was performed as above. Reverse transcriptase (RT) sequencing reads were aligned to hs38DH. This brute force alignment of spliced products to a genomic reference was a conservative approach designed to reduce potential alignment artifacts due to variable splicing, and focus on exonic sequences. Mutations called from DCS-corrected reads and uncorrected reads were compared.

### Mutation mix and negative control

To demonstrate the sensitivity, specificity, accuracy and precision of Duplex Sequencing, a mutation standard was prepared and Duplex Sequenced in replicate library preps. Four relapse DNA samples with TKI-R mutations, 1 pre-TKI DNA sample with a TKI-R mutation, and the Horizon Myeloid DNA Standard (Horizon Discovery) were diluted into control DNA derived from the peripheral blood of a healthy 18-year old, male, non-smoking donor. The resulting DNA mixture had 9 unique *ABL1* mutations at different predicted frequencies. Ten replicate 500 ng libraries each of the mutation mix or the pure control DNA were prepared and analyzed. As Duplex Sequencing uniquely tags every DNA molecule, and because each library originated from discrete DNA molecules, Duplex Sequencing data from technical replicates was aggregated to increase total molecular depth. Background mutation frequency was calculated by first masking germline SNPs, and then dividing total non-reference allele counts (numerator) by total Duplex bases sequenced (denominator) across all nucleotide positions in *ABL1* exons 4–10.

### Assessment of TKI resistance mutations

Mutations were classified as conferring TKI resistance as previously reported^16^. To determine if the number of TKI resistance mutations vs. other mutations was significantly enriched, a null model was computed. Briefly, across *ABL1* exons 4–10, all possible single-nucleotide substitutions were simulated. The null TKI resistance mutation rate was the number of simulated mutations annotated as conferring TKI resistance divided by the total number of simulated mutations. A Bonferroni correction was carried out to account for multiple comparisons.

### Mutation spectra

Nucleotide spectra were calculated for each sample by enumerating the number of each type of single-nucleotide mutations observed. Mutations were first converted to pyrimidine space and then each type of mutation was counted (C > A, C > G, C > T, T > A, T > C, T > G). The proportion of each mutation type was the normalized fraction of mutations over the total number of mutations.

### Sanger sequencing

Mutation analysis for ABL1 KD was performed using a nested PCR approach followed by Sanger sequencing. The initial round of BCR-ABL1 amplification (for transcripts e1a2, b2a2 and b3a2) from cDNA samples was followed by 2 separate PCR reactions spanning codons 221–380 and 350–500 of the ABL KD as previously published^17,18^. Following purification, PCR products underwent standard dideoxy chain-termination DNA sequencing using M13 tagged primers and Big Dye chain terminator reagents with ABI PRISM 3100/3700 genetic analyzers. The results were evaluated using SeqScape and Sequence Analysis software (ABI, Foster City, CA). The mutations were determined by comparing the forward and reverse sequences of the samples the unmutated ABL sequence. The sensitivity of the assay was 10 to 20% mutation-bearing cells in the analyzed population as estimated by dilution studies.

### Statistical methods

Patient characteristics were summarized using median (range) for continuous variables and frequencies (percentages) for categorical variables. Associations between categorical and continuous variables were assessed using chi-square tests and one-way analysis of variance. Concordance of predicted vs. observed VAFs were assessed using Pearson correlation calculation. To test if the number of mutations observed in a single sample was significantly above background a two-tailed binomial test was applied. All statistical tests were performed using GraphPad Prism 6.

## Results

### Patient characteristics

The median age of the cohort was 57 years (range, 20–80 years). The TKI received was imatinib in 7 patients (11%), dasatinib in 35 patients (56%), and ponatinib in 21 patients (33%). Four patients were not evaluable for response (early death, *n* = 3; lost to follow-up, *n* = 1). Of the 59 patients evaluable for response, all achieved complete remission (CR). CR was achieved after one cycle in 57 patients and after two cycles in two patients. Thirty-three of 50 evaluable patients (66%) achieved complete molecular remission (defined as absence of a quantifiable BCR-ABL1 transcript by PCR) within 3 months of therapy, and 45 of 58 patients (78%) achieved complete molecular remission at any time point. Twelve patients (19%) received allogeneic hematopoietic stem cell transplant in first remission. With a median follow-up of 54.3 months, 13 patients relapsed. The median relapse-free survival for the entire cohort was 52.8 months and the median overall survival was 76.1 months.

### Pretreatment *ABL1* KD mutations and association with clinical outcomes

**Fig. 1:**
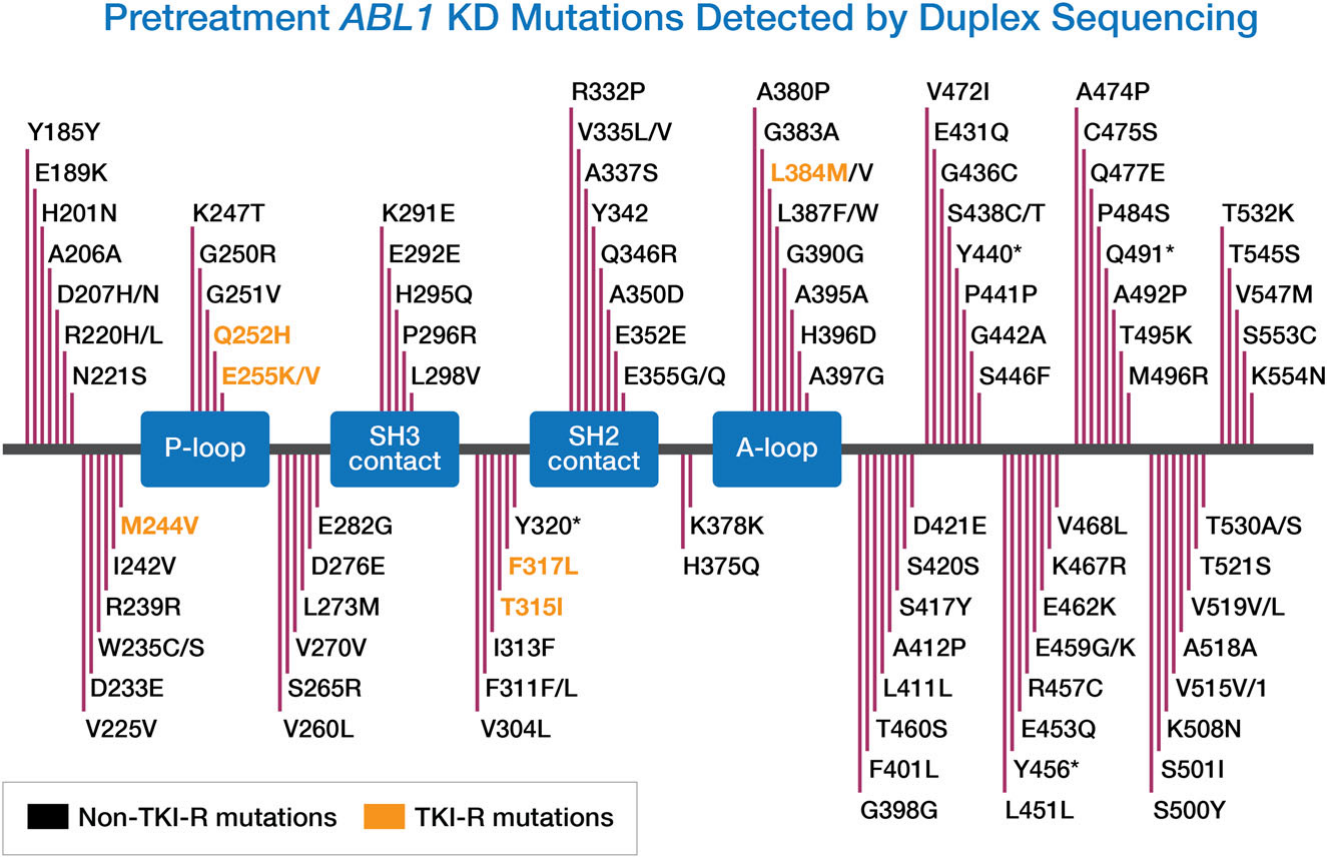
Pretreatment *ABL1* KD mutations detected by Duplex Sequencing. A total of 129 pretreatment *ABL1* mutations were detected. Seven unique resistance mutations (M244V, Q252H, E255K, E255V, F317L, T315I, and/or L384M) were detected in ten patients and are shown in yellow. In total, 1.74 billion Duplex base-pairs were generated, with an average of 27.65 million base-pairs per sample. The median Duplex depth was 12,698 × (range, 4458×−24,507×). Asterisks indicate stop codons.

In total, 1.74 billion Duplex base-pairs were generated from pretreatment gDNA using Duplex Sequencing, with an average of 27.65 million base-pairs per sample. The median Duplex depth across the *ABL1* KD was 12,698×(range, 4458×−24,507×). A total of 129 pretreatment *ABL1* mutations were detected by Duplex Sequencing, with ≥ 1 mutation detected in 49 patients (78%) (Fig. 1). However, these mutations were generally present at very low levels (median VAF 0.008% [range, 0.004%–3.71%] and median mutant allelic depth was only one count [range, 1–533]). The VAF was ≥ 0.01% for 45 mutations (35%) and ≥ 0.1% for only 6 mutations (5%). One hundred and four of these mutations (81%) were missense mutations, 5 (4%) were nonsense and 20 (16%) were synonymous mutations. The median number of independent mutations per patient was 2 (range, 0–8). Of the 129 pretreatment mutations, only 16 (12%) were identified that have been reported to confer resistance to at least one BCR-ABL1 TKI^16,19,20^. Table 1 shows the 16 TKI-R mutations detected across ten patients. The median VAF of TKI-R mutations was 0.02% (range, 0.006%–3.71%) and median mutant allelic depth was only 1 count (range, 1–533). Other than 1 TKI-R mutation (F317L) at 3.71%, all others were < 1%. Notably, only a single instance of a T315I mutation was detected and this was present in a single molecule from one sample. No patients with pretreatment TKI-R mutations detected by Duplex Sequencing subsequently relapsed, despite 8 of these patients harboring a TKI-R mutation known to be at least moderately resistant to the TKI they received. There was no difference in the number of pretreatment *ABL1* mutations between patients who relapsed and those who did not (median number of mutations: 2 [range, 1–4] and 2 [range, 0–8], respectively; *P* = 0.65).

**Table 1:** Characteristics of patients in whom ≥ 1 pretreatment TKI resistance mutation was detected by Duplex Sequencing.

Patient	TKI received	Pretreatment *ABL1* KD mutation(s)	VAF (%)	Anticipated sensitivity of mutation to TKI received
#1	Imatinib	T315I	0.009	Highly resistant
#2	Dasatinib	M244V	0.015	Sensitive
#3	Dasatinib	F317L	0.008	Resistant
#4	Dasatinib	E255K	0.006	Resistant
#5	Dasatinib	F317L	0.007	Resistant
#6	Dasatinib	L384M	0.065	Moderately resistant
		M244V	0.080	Sensitive
		Q252H	0.166	Moderately resistant
#7	Dasatinib	E255V	0.008	Moderately resistant
		E255K	0.023	Resistant
		F317L	0.006	Resistant
#8	Dasatinib	E255K	0.043	Resistant
		F317L	3.712	Resistant
#9	Ponatinib	F317L	0.374	Sensitive
#10	Ponatinib	E255K	0.015	Resistant
		M244V	0.027	Moderately resistant

Sensitivities are based on in vitro data (adapted from Redaeeli et al.^16^).

*TKI* tyrosine kinase inhibitor, *KD* kinase domain, *VAF* variant allelic frequency.

Sanger sequencing was performed at the time of relapse on 10 of the 13 patients who relapsed. TKI-R mutations were detected at relapse in 6 patients (60% of evaluable relapses). The TKI-R mutations detected were T315I in three patients, and F317I, V299L and V338G in one patient each. None of these relapse mutations was detected by Duplex Sequencing in pretreatment samples, further supporting a lack of association between the ultra-low frequency pretreatment mutations detected by Duplex Sequencing and clinical outcomes.

### Sensitivity and specificity of Duplex Sequencing

**Fig. 2:**
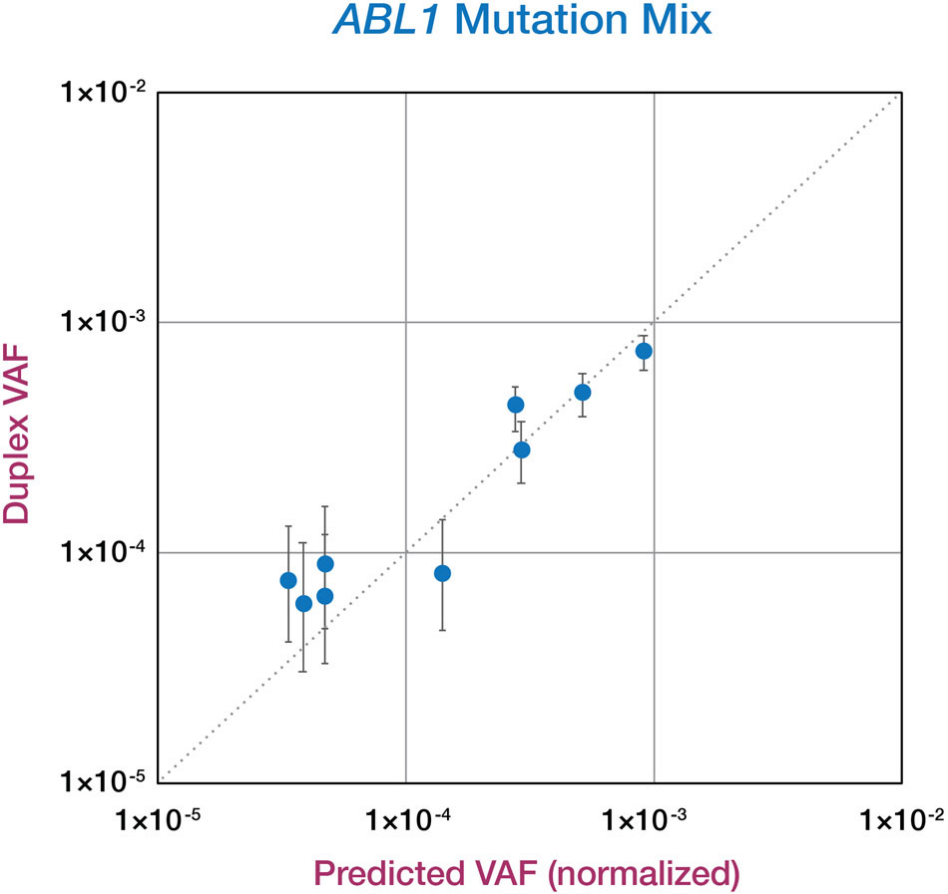
Association of predicted vs. detected *ABL1* mutation VAFs. Predicted VAF (*x*-axis) was calculated by multiplying the original sample VAF by the dilution factor. To account for differences in performance due to varying DNA quality of individual samples in the mix, predicted VAFs were normalized to mean Duplex depth of individual samples vs. the negative control DNA preparations. The *y*-axis represents observed Duplex VAF of each mutation in the mix. Error bars represent Wilson 95% confidence intervals. *r*^2^ for observed vs. expected VAF = 0.93.

**Fig. 3:**
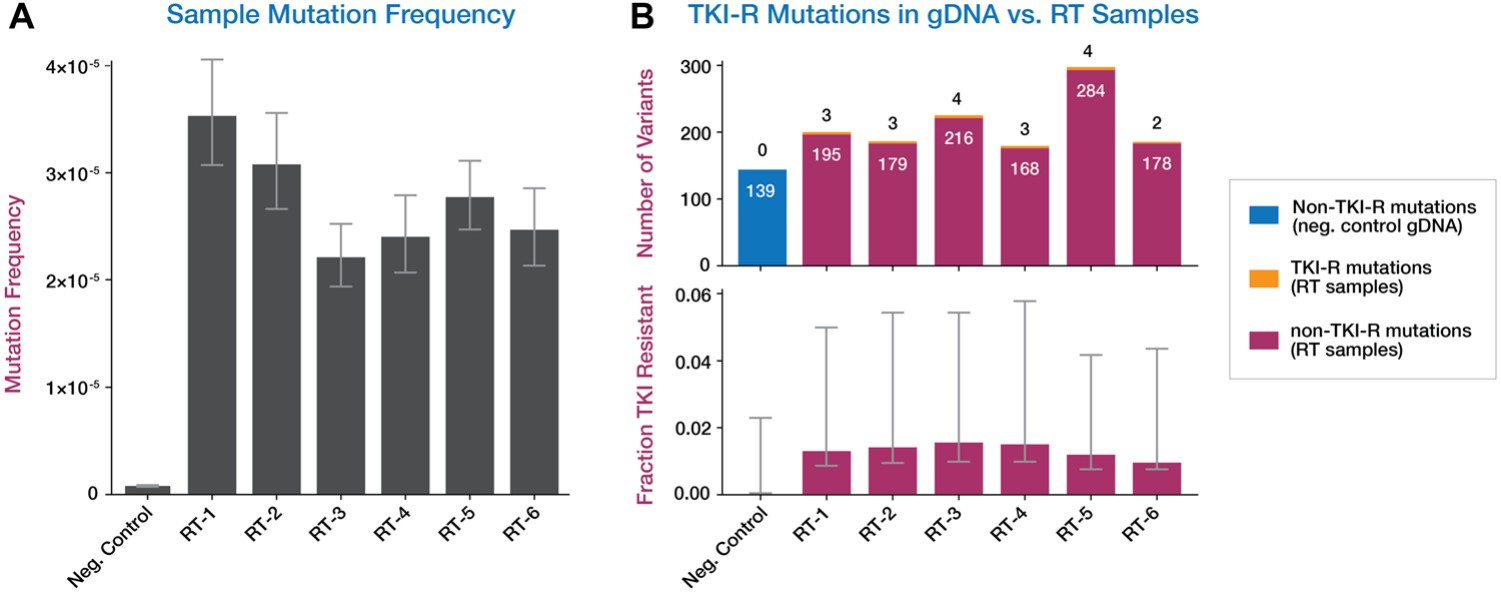
Comparison of Duplex Sequencing on gDNA vs. cDNA produced by RT-PCR. **a** Mutation frequency (defined as total non-germline alternate allele counts divided by total Duplex bases sequenced) of gDNA from the negative control vs. six RT products from patients with no *ABL1* mutations detected by Duplex Sequencing in pretreatment gDNA. Data represent single preps for the RT samples or the average of ten replicate preps for the negative control. Error bars represent the 95% Wilson confidence interval (CI). **b** Number of TKI-R and non-TKI-R variants detected in genomic DNA and in six RT products from patients with no *ABL1* mutations detected by Duplex Sequencing at baseline. In the top panel, genomic DNA non-TKI-R mutations are shown in blue, TKI-R mutations in RT samples are shown in yellow, and non-TKI-R mutations in RT samples are shown in pink. In the bottom panel, error bars represent 95% CI for the fraction of TKI-R variants out of total variants per sample.

To validate the Duplex Sequencing methodology, we examined its sensitivity and specificity using dilution experiments of nine different single-nucleotide variants (SNV) in *ABL1*, including six TKI-R mutations, from primary patient samples and cell line standards. Samples were mixed into control gDNA (derived from peripheral blood of a healthy 18-year-old) for predicted VAFs ranging from 1/250 to 1/25,000. Mean Duplex molecular depth of 158,823x was generated for the mutation mix. Mutations were identified by Duplex Sequencing with 100% sensitivity down to VAF < 5.6 × 10^–5^, with high precision (*r*^2^ = 0.93 for linear correlation of detected vs. predicted VAF) (Fig. 2). Duplex sequencing of a pure sample of the control DNA yielded a mean Duplex molecular depth was 139,819x. None of the spiked-in mutations was detected in the negative control (100% specificity). Background mutational frequency (defined as total non-germline alternate allele counts divided by total Duplex bases) was 5.35 × 10^–7^ (Fig. 3a), which is approximately one mutation per 2 million Duplex base-pairs. No TKI-R mutations were detected in the control, out of 496,167,962 total Duplex sequenced base-pairs.

### Comparison of accuracy of Duplex Sequencing on gDNA and RT-PCR products

As previous studies reporting low-level pretreatment TKI-R mutations have predominantly relied on RT-PCR as part of analysis, we sought to evaluate whether introduction of errors during the RT process could account for our contrary finding that pretreatment TKI-R mutations are both exceptionally rare and not associated with relapse in Ph + ALL. RT-PCR was used to generate first-strand cDNA followed by targeted second-strand synthesis from blood or bone marrow RNA from six patients in whom no pretreatment *ABL1* KD mutation was detected by Duplex Sequencing in gDNA. Duplex Sequencing was then performed on the RT-produced cDNA and compared to that of gDNA from a young healthy control. Even with Duplex error-correction, the overall mutation frequencies in cDNA products were between approximately 2–4 × 10^–5^ (in agreement with the reported MMLV RT polymerase error rate^11,21^), and ~50 times higher than the mutational frequency observed in the corresponding control gDNA sample (Fig. 3a). All RT-produced cDNA samples had false-positive TKI-R mutations (Fig. 3b). At a median Duplex depth of 10,510x (range, 8423x–10,357x), cDNA samples had 2–4 Duplex error-corrected TKI-R variants per sample, with a median VAF of 0.013% (range, 0.008%–3.319%). There were no TKI-R mutations detected in the negative control gDNA, despite Duplex Sequencing to >10 times greater depth relative to the RT samples. TKI-R mutations in cDNA were not significantly enriched above a null model of all possible TKI-R mutations vs. all possible coding mutations. A relative excess of C > T and T > C nucleotide changes was observed in the RT-generated cDNA products, accounting for 78.4% of observed mutations, which is consistent with previously reported RT-generated mutation spectra^10,11^. In contrast, mutations in the negative control gDNA were predominantly C > A nucleotide substitutions (66.7% of observed mutations), whereas only 13.1% were C > T or T > C.

### Evolution of resistance during TKI therapy

**Fig. 4:**
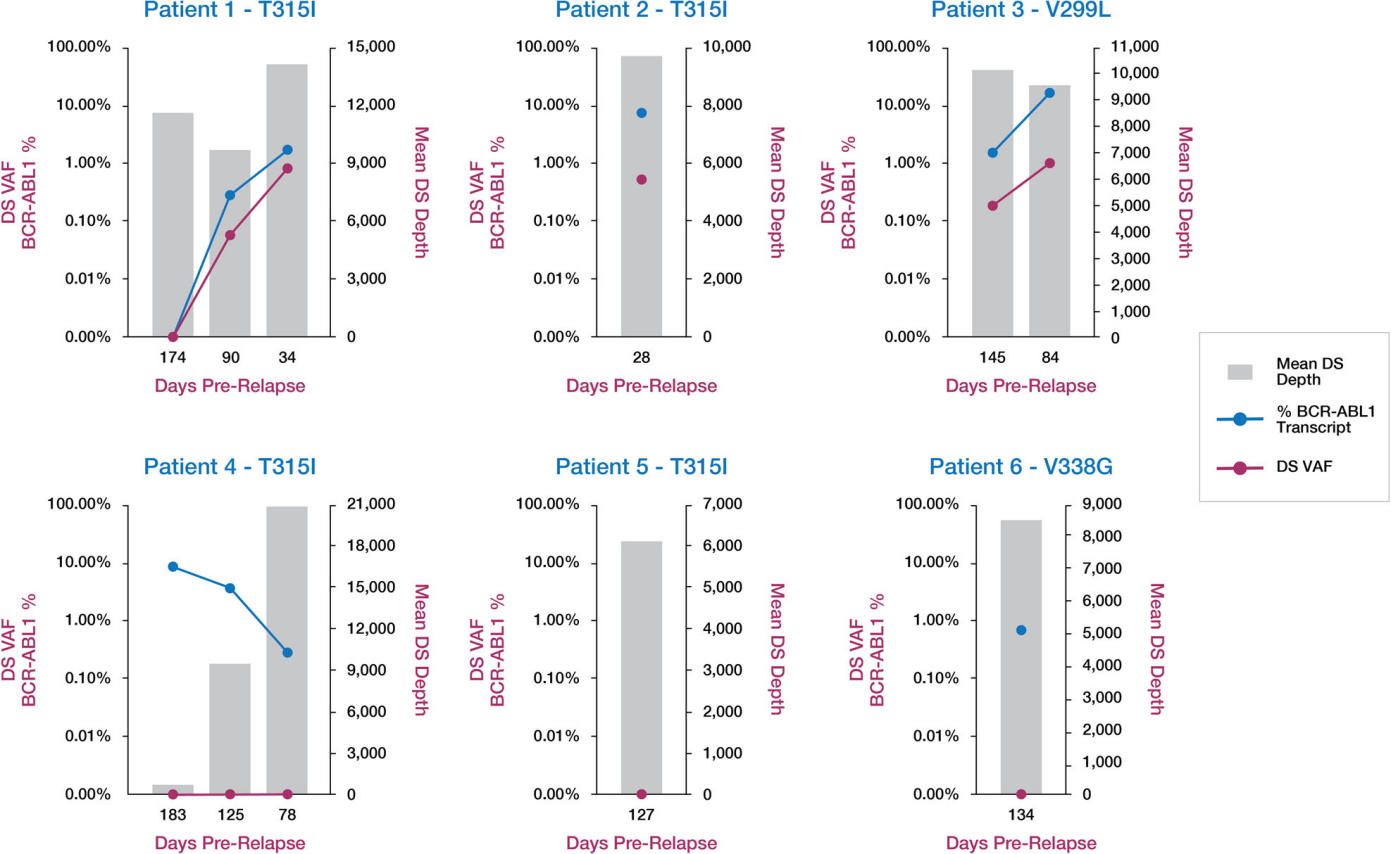
TKI-R mutation detection in pre-relapse TKI-treated patients. Duplex Sequencing (DS) VAF of relapse mutation (red) and *BCR-ABL1* transcript % (blue) are plotted on the primary *y*-axis, along with mean Duplex depth (gray bars, secondary *y*-axis) for 12 total DNA samples from pre-relapse time points from six patients who relapsed with a TKI-R mutation. Sanger sequencing of relapse DNA identified T315I in four patients, and V299L and V338G in one patient each. A quantifiable pre-relapse *BCR-ABL1* transcript was unavailable for patient 5. Duplex Sequencing did not identify V338G in relapse or pre-relapse DNA from patient 6.

We hypothesized that although TKI-R mutations are rare prior to TKI therapy, they may be detectably enriched after initiation of therapy but prior to overt relapse due to natural selection conferred by TKI exposure. Duplex Sequencing was performed on 12 bone marrow DNA samples from six patients on TKI therapy who relapsed with a TKI-R mutation, collected at time points up to 6 months prior to relapse. Pre-relapse samples generated peak Duplex depths up to 23,094x (median 10,600x; range 838x–23,094x). Other than the sample with the least DNA available (only 123 ng of DNA sequenced), all other samples generated peak Duplex depth of 5891x or higher (Fig. 4).

In stark contrast to pretreatment samples, we identified the relapse TKI-R mutation up to 145 days prior to relapse in 5 DNA samples from 3 of 6 patients at VAFs ranging from 0.06%–0.91% (Fig. 4). For the three patients where the relapse TKI-R mutation was not detected at an earlier time point, one patient had a Sanger-reported mutation at time of relapse that was not validated by Duplex Sequencing on the relapse DNA, one patient did not have a quantifiable *BCR-ABL1* transcript in the pre-relapse sample, and one patient showed steadily declining *BCR-ABL1* transcript % from 183 to 78 days prior to relapse. Given the extremely low background frequency, the sensitivity of Duplex Sequencing in gDNA is determined primarily by the Duplex molecular depth, which is limited by initial DNA input. For example, at 10,000x Duplex depth, the lowest possible VAF will be 1 × 10^–4^. Given the Duplex depth of the remaining seven pre-relapse DNA samples in which no mutation was detected by Duplex Sequencing (Fig. 4), TKI-R mutations, if present, are below frequencies of 8.6 × 10^–5^, 4.8 × 10^–5^, 1.1 × 10^–4^, 1.0 × 10^–4^, 1.4 × 10^–3^, 1.9 × 10^–4^, 1.6 × 10^–4^, and 1.2 × 10^–4^.

## Discussion

We applied highly sensitive and specific Duplex Sequencing to samples from patients with Ph + ALL in order to gain insights into the landscape of *ABL1* mutations, both prior to treatment and in response to selective pressure from TKI exposure. Although exceptionally low-level mutations were detectable prior to TKI therapy, 88% of these mutations have not been previously reported to confer TKI resistance, suggesting that most of these are random mutation events occurring as part of the normal aging process. Even among the few *ABL1* TKI-R mutations that were identified by Duplex Sequencing, there was no association with relapse. Conversely, among patients who relapsed with *ABL1* mutations, these mutations were not detected by Duplex Sequencing of pretreatment samples. Taken together, these findings suggest that pretreatment *ABL1* mutations do not represent clinically meaningful subclones that contribute to relapse. The detection of pretreatment *ABL1* mutations is therefore unlikely to meaningfully guide upfront treatment decisions (e.g., TKI selection) in patients with newly diagnosed Ph + ALL.

The development of *ABL*1 KD mutations is the dominant mechanism of resistance in patients with Ph + ALL, with T315I mutations detected in up to 75% of patients at the time of relapse after treatment with a first- or second-generation BCR-ABL1 TKI, neither of which have clinical activity against these T315I mutations^2,5,6^. Ponatinib is a third-generation pan-BCR-ABL1 TKI that is active against nearly all *ABL1* KD mutations, including the T315I mutation^22^. With the combination of intensive chemotherapy and ponatinib, a 5-year OS rate > 70% has been reported, which compares favorably to historical data with first- or second-generation TKIs^14,23^. The lack of association between pretreatment TKI-resistant *ABL1* mutations and subsequent relapse observed in our study suggests ponatinib-containing regimens exert superior activity in newly diagnosed Ph + ALL primarily by preventing later outgrowth of random, background levels of resistance mutations, rather than suppressing meaningfully subclonal, pretreatment *ABL1* mutations. Owing to the dearth of clinically relevant TKI-resistance mutations detectable at diagnosis even with the highest sensitivity sequencing method available, our findings do not support the routine use of pretreatment *ABL1* mutation analysis to personalize TKI selection in patients with newly diagnosed Ph + ALL.

Our findings that pretreatment TKI-R mutations are relatively rare in Ph + ALL (detected in 16% of patients in our cohort) and do not contribute to relapse are contrary to several reports from other investigators^6–9^. For example, the EWALL group detected *ABL1* T315I mutations in 10 out of 43 older patients with newly diagnosed Ph + ALL using allele-specific PCR^6^. In contrast, despite a demonstrated sensitivity of at least 5.6 × 10^–5^ (the lowest frequency mutation in our DNA mixture) with Duplex Sequencing, we detected only one instance of a T315I mutation in a single molecule from one pretreatment sample. The discrepancy between our findings and those of others who have reported much higher rates of TKI-R mutations is likely due to differences of methodology for mutation assessment. Previous studies have generally relied on RT-generated cDNA for analysis, which definitively introduces errors, leading to false positives^10,11^. The demonstration approach we undertook in this study, in fact, likely represents a low end of RT-PCR background mutation frequency; whereas we performed a single cycle of targeted second-strand synthesis prior to Duplex Sequencing library preparation, other reports describe up to 50 cycles of PCR prior to library prep or other analysis^6–9^. Without Duplex error-correction, the number of PCR-induced false-positive TKI-R mutations will be much higher than observed here as well.

In our study, we observed an ~50-fold difference in mutation frequency when Duplex Sequencing was applied to gDNA vs. RT-generated cDNA. Interestingly, 0 counts of TKI-R mutations were identified in the negative control while every RT sample had false-positive TKI-R mutations. This may be explained in part by the biased mutation spectra of RT, which predominately leads to C > T and T > C nucleotide changes, and which we observed when Duplex Sequencing was performed on RT-generated cDNA^10,11^. In contrast, when Duplex Sequencing was applied to gDNA, most nucleotide changes were C > A substitutions, which are commonly observed in 8-oxo-guanine-mediated age-associated mutagenesis. This further supports the conclusion that the small number of pretreatment *ABL1* mutations observed in our study were largely the result of random mutagenesis rather than an actively evolved form of pre-existing resistance, and are clinically inconsequential^24^. These findings suggest that RT-based methods are likely not sufficiently accurate to identify low-level mutations and, therefore, should not be used to guide prognostic assessment or therapeutic decision-making at any point in the disease course other than at the time of overt relapse. This conclusion in agreement with a recent report in which pretreatment T315I mutations were detected by digital droplet RT-PCR in approximately one-quarter of adults with newly diagnosed Ph + ALL, although only one patient was confirmed to have an expanded T315I clone in later follow-up^25^. In this study and others, it is likely that many (if not all) of these detected pretreatment T315I mutations were artefacts stemming from the RT process, which likely explains the lack of subsequent clonal expansion of the TKI-R mutation.

The superior accuracy of Duplex Sequencing compared to RT-PCR has broad clinical implications, not only in Ph + ALL, but also in the detection of low-level mutations across cancers, which may allow for early interventions to target emerging resistant subclones^26^. In pre-relapse samples from patients undergoing TKI therapy, Duplex Sequencing identified the relapse mutation in 3 of 6 patients up to 5 months prior to relapse at VAF down to 0.06%. These pre-relapse *ABL1* KD mutations (T315I in two patients and V299L in one patient) were potentially actionable, as switching to a broader spectrum TKI such as ponatinib could have theoretically suppressed these emerging resistance clones. In the remaining three patients either the relapse mutation was not verified by Duplex Sequencing, the *BCR-ABL1* transcript was not quantifiable, or *BCR-ABL1* transcripts were decreasing in time points leading up to relapse. Mutation detection power was severely limited by minimally available DNA from dried bone marrow aspirate smears from pathology archives, which were the only samples available for this analysis. Duplex Sequencing data yield is dependent on input DNA quantity (DNA mass comprising unique genome copies) and quality (amplifiable double-stranded DNA molecules). In future studies, DNA from fresh-frozen blood or bone marrow of patients with ongoing TKI exposure would likely markedly increase sensitivity.

In summary, we empirically demonstrated Duplex Sequencing to have 100% sensitivity down to a level of 0.005% with 100% specificity. Using this technology, we identified very low-level *ABL1* KD mutations in > 75% of patients with newly diagnosed, previously untreated Ph + ALL; however, not a single KD mutation in any patient at time of diagnosis contributed to relapse, suggesting that the majority of these pretreatment mutations are simply due to random, age-related mutagenesis. The excellent accuracy of Duplex Sequencing has broad implications in the detection of low-level mutations not only in leukemias but across cancers, with the potential for early intervention with targeted therapies based on detection of emerging resistant subclones. Future studies using this technology will be needed to quantify the benefit of prospective monitoring and early intervention in such cases.

## Supplementary information

Reproducibility checklist
